# Association of Obesity and Thyroid Cancer at a Tertiary Care Hospital in Pakistan

**DOI:** 10.7759/cureus.2364

**Published:** 2018-03-26

**Authors:** Adnan Ali, Yumna Mirza, Urooj Faizan, Nida Zahid, Muhammad S Awan

**Affiliations:** 1 Department of Surgery, Aga Khan University Hospital, Karachi, PAK

**Keywords:** bmi, endocrinology, oncology

## Abstract

Objective

Thyroid cancer (TC) is one of the most common endocrine malignancies with a rapidly increasing worldwide presence. In Pakistan, it is more prevalent in females than males and has an incidence rate of 2.1%. Obesity and excess body mass index (BMI) has been linked to several cancers and is thought to be a risk factor for TC. We aim to investigate the incidence of TC in our population and understand it’s correlation with obesity.

Subjects

The study was a retrospective case series conducted in the years 2000 to 2014, at the Aga Khan University Hospital (AKUH), Karachi, Pakistan where 156 patients who had been diagnosed and treated for TC were analyzed. Clinicopathological data was collected from medical records of these patients and weight and height were measured, pre-surgery, post-surgery, and at follow up. The BMI was correlated with patient variables for any significant associations.

Results

The patient set comprised of 38.5% males and 61.5% females with a mean age of 47.77 (SD ± 14.35). The BMI was significantly associated with age as 72.8% of participants were obese and >45 years old as compared to 27.2% who were under 45 years and obese (p-value <0.001). Upon comparison of the pre-surgery, post-surgery, and current mean BMI, Bonferroni pairwise comparisons showed no significant difference (p>0.999).

Conclusion

The majority of-of TC patients among the Pakistani population were obese and female. Age was significantly associated with the risk of having a higher BMI. Moreover, differences in BMI pre and post-surgery could not be statistically proven.

## Introduction

Thyroid cancer (TC) is the most common endocrine malignancy and its incidence is increasing dramatically all over the world since the last few decades [1]. In the United States, the incidence has increased from 3.6 per 100,000 in 1973 to 8.7 per 100,000 in 2002 [2] and it has been ranked the 5th most common cancer in women with nearly 3% cancer diagnosis in 2010 (45,000 new cases). In Pakistan, the incidence rate of TC is 2.1% among all malignancies and it was found to be more prevalent among females with a female to male ratio of 2.6:1 [3] The reason for this significant rise in incidence can be due to recent advanced screening techniques and early detection rather than true increase in disease occurrence, but still the exact cause remains controversial [4].

Thyroid cancer is a malignancy arising from an abnormal cell cycle of follicular or parafollicular thyroid cells and it is categorized into differentiated TC (DTC) and undifferentiated TC. DTC accounts for approximately 90% of all thyroid malignancies and consists of three different histological subtypes: papillary (two-third cases), follicular (10%-20%), and medullary (5%-10%) [5]. Undifferentiated TC i.e., anaplastic type accounts for less than 5% cases of all TC. In Pakistan, among all variants of TC, papillary carcinoma was found to be the most common (69%), followed by follicular carcinoma (11.6%), medullary carcinoma (6.9%), anaplastic carcinoma (5.9%), non-Hodgkin’s lymphoma (2.9%), and unclassified tumours (0.9%) [3] and this rate of occurrence is comparable to international data.

The sexual disparity in TC incidence suggests that there may be some hormonal and reproductive factors responsible. Irregular menstrual cycle, miscarriages or abortions, multiple pregnancies or live births, surgical menopause which includes hysterectomy and bilateral oophorectomy and lactation suppressants were all associated with TC risk among women. However, miscarriage or abortion, particularly in the first pregnancy, is the most established contributing reproductive factor. The most common risk factor was found to be previous exposure to ionizing radiations, benign thyroid conditions which carry the risk of becoming malignant, and iodine deficiency [6]. Thyroid nodular disease is one of the well-established independent risk factors for the development of TC and its incidence increases with increasing weight (>60 kg) and increasing age, specifically in women [7].

According to many studies, environmental and genetic predispositions play a major role in TC development. Furthermore, obesity is a growing health problem not only in developed countries but all over the world. Obesity is precisely defined as body mass index (BMI) greater than 30 kg/m2 and it carries an increased risk for many chronic illnesses such as hypertension, diabetes, heart diseases, sleep apnoea, musculoskeletal diseases, and cancers at several organ sites. It has been determined that about 20% of all cancer cases are due to excess bodyweight [8].

Excess BMI has also been linked to aggressiveness of the tumor, severe presentation, and higher risk of death as it has been shown by studies that obese patients tend to present with more advanced stages of breast cancer [9], higher grade of prostate cancer with higher risk of recurrence [10] and tumor size with higher overall mortality risk from all cancers combined.

Like other cancers, obesity moderately increases the aggressiveness of TC as well as the rate of microscopic extra thyroidal invasion and advanced TNM stages, the majority presenting with stage III and IV [11]. However, there have been other studies showing no relation of TC with excess BMI. In a recent study conducted in China, it was found that the prevalence of thyroid nodules in both the sexes with an overall detection rate of 42.6% with 40.0% and 46.5% among men and women respectively, while TC was reported in 0.3% overall and 0.15% in men and 0.50% in women. The detection rate was directly related to increasing age, high systolic blood pressure, lighter weight, and smaller height [12]. Similarly, another study conducted in San Francisco also reported no association of TC with height, weight, and BMI [13].

As a result, it can be grasped that the relationship between BMI and TC is a controversial one, as some studies have quoted positive relation, particularly in females, while others have not. Thus the aim of our study was to investigate the association of BMI and TC in the Pakistani population with the hypothesis that increasing BMI can be a significant factor for thyroid malignancies.

## Materials and methods

The study was of cross-sectional design in which a total of 221 patients who had been diagnosed with and subsequently treated for TC (either complete thyroidectomy or partial thyroidectomy) in the years 2001- 2014 at Aga Khan University Hospital (AKUH), Karachi, Pakistan were identified and then further screened according to the inclusion/exclusion criteria. The diagnosis was made on the basis of preoperative fine needle aspiration cytology (FNAC) and was confirmed by postoperative frozen section biopsy of the specimen. An ethical exemption was sought from the Ethical Review Committee of AKUH.

Out of the pool of 221 patients, a total of 156 patients were included in the study based on the fact that they had provided informed consent, had complete clinicopathologic data, and known follow up status. Similarly, patients were excluded if they lacked any of the above. Survival status was defined as whether the patients were alive or dead at last contact (through the clinic or follow up visit). For the calculation of BMI, the following formula was used:

BMI = body weight in kg/ height in m^2 ^[14].

Body mass index or Quetelet index is a measure of the relative size of the body and it is defined as body mass in kilograms (kg) divided by the square of height in meters (m^2^) [14]. The World Health Organization (WHO) regards a BMI to be normal (18.5 to 25 kg/m^2^), underweight (<18.5 kg/m^2^), overweight (>25 kg/m^2^), obese (>30 kg/m^2).^ In Asians, the cut-off values of overweight and obesity are lower than the WHO criteria. Proposed classification of weight by BMI in adult Asians is found to be underweight (<18.5), normal (18.5 to 22.9), overweight (>23), and obese (>25) [15].

Statistical analysis

We performed the analysis using Statistical Package for the Social Sciences (SPSS), version 22.0 for Windows (SPSS Inc., Chicago, IL, US). Descriptive statistics were calculated for categorical variables by computing their frequencies and were assessed using χ2 test. The distribution of quantitative variables was computed by their means and standard deviations were reported after checking for normality assumption and assessed using independent t-test. The pre and post-BMI difference of each individual were considered by paired t-test and the difference in pre, post, and current BMI of each individual was assessed by repeated measure analysis of variance (ANOVA). Bonferroni pairwise comparison test was applied to assess the pairwise difference. Cross-tabulation was run for each categorical variable to look for any zero cell count or sparse data and such variables were grouped together in a meaningful way.

## Results

The patient set comprised of 60 (38.5%) males and 96 (61.5%) females, making up a total of 156 patients with a mean age of 47.77 (SD ± 14.35). Out of these, the majority were above 45 years of age (57.4%) and were obese (51.9%) (Table 1).

**Table 1:** Patient characteristics (n=156) BMI: body mass index.

		Frequency (%)
Age division	<45 years	66 (42.9)
	≥45 years	89 (57.1)
Gender	Male	60 (38.5)
	Female	96 (61.5)
Preoperative BMI	Underweight	8 (5.13)
	Normal weight	46 (29.5)
	Overweight	21 (13.5)
	Obese	81 (51.9)
Postoperative BMI	Underweight	10 (6.4)
	Normal weight	39 (25)
	Overweight	29 (18.6)
	Obese	78 (50)
Current BMI	Underweight	10 (6.4)
	Normal weight	31 (19.9)
	Overweight	26 (16.7)
	Obese	89 (57.1)
Type of tumour	Papillary	115 (73.7)
	Medullary	16 (10.3)
	Follicular	11 (7.1)
	Anaplastic	5 (3.2)
	Others	9 (5.8)
Type of surgery	Complete thyroidectomy	112 (71.8)
	Partial thyroidectomy	35 (22.4)
	Unknown	9 (5.8)
Survival status	Alive	147 (94.2)
	Dead	9 (5.8)

Categorizing the patient set into groups above and below the age of 45 years revealed that the majority of males were obese in both age groups. However, the larger part of females under 45 were of normal weight and obese over the age of 45. About 69% of females >45 years of age were obese versus 31% of women <45 years of age (Table 2).

**Table 2:** Gender and age-specifc body mass index (BMI) values

Age	BMI	Gender n(%)	
		Male	Female
<45 years	Underweight	3 (12.50)	3 (7.10)
	Normal weight	8(33.3)	18 (42.9)
	Overweight	4(16.7)	8(19)
	Obese	9(37.5)	13(31.0)
>45 years	Underweight	1(2.8)	1(1.9)
	Normal weight	12(33.3)	8(15.10)
	Overweight	1(2.8)	7(13.2)
	Obese	22(61.1)	37 (69.80)

Moreover, this distinction of age was significantly associated with a higher BMI as 72.8% of participants were obese and >45 years old as compared to 27.2% who were under 45 years and obese (p-value <0.001) (Table 3).

**Table 3:** Correlation of body mass index (BMI) with age p-value calculated using chi-square test and considered significant at <0.05.

Age	BMI n (%)				P value
	Underweight	Normal weight	Overweight	Obese	<0.001
<45 years	6 (75)	26(56.5)	12 (60)	22(27.20)	
≥45 years	29(25)	20(43.5)	8(40)	59(72.8)	

Upon comparison of pre-surgery, post-surgery, and current mean BMI (which was collected at last follow up visit), Bonferroni pairwise comparisons showed no significant difference (p>0.999) (Table 4). However, the difference in mean post and mean current BMI was marginally significant (p=0.054). Furthermore, no mean BMI difference was observed between the two genders in preoperative (p=0.59), postoperative (p=0.28), and current BMI (p=0.09).

**Table 4:** Comparison of mean preoperative, postoperative, and current body mass index (BMI)

BMI	Preoperative BMI n(%)	Postoperative BMI n(%)	Current BMI n(%)
Underweight	8(5.13)	10(6.4)	10(6.4)
Normal weight	46(29.5)	39(25)	31(19.9)
Overweight	21(13.5)	29(18.6)	26(16.7)
Obese	81(51.9)	78(50)	89(57.1)
Mean BMI ± SD	25.28 ± 4.51	25.23 ± 4.58	26.11 ± 5.05

## Discussion

Several studies have shown links between increased BMI and high prevalence of TC in different parts of the world [16-17]. The relation of obesity and risk of cancer has been studied thoroughly as obesity is becoming a worldwide health challenge. The trends of increasing obesity also impact the incidence, mortality, and morbidity rates of the majority of cancers. Our study reflects the growing trend of obesity as the majority of patients enrolled were 45 years or older and were also obese, having BMI >25.

Several studies have shown the association of excess BMI with the risk of cancer at several organ sites including colon, breast (in postmenopausal women), endometrium, oesophagus, and kidney [18]. In recent decades, the incidence rate of TC has risen dramatically along with a significant rise in obesity rates [1]. But this relation is controversial because several studies have suggested a positive relation of BMI with TC [11,19] while many studies have shown no significant relation [20].

This study revealed that the majority of males suffering from TC were obese, whether under or over the age of 45 years. In contrast, female patients of TC were mostly of normal weight if under the age of 45 years, and overweight if they were above 45 years. Since women above the age of 45 typically experience menopause, the association of menopause and TC has been a topic of interest. A systematic review and meta-analysis conducted in 2015 concluded from the data of 25 independent studies that common hormonal factors such as oral contraceptives and hormone replacement therapy did not have an effect on TC risk. However, an older age at menopause and parity were considered risk factors for TC, with a longer duration of breast feeding providing a protective effect [21].

An important observation was that a higher age was significantly associated with an increased BMI with a p-value of <0.001. A pooled analysis of 12 case-control studies showed a significant association of BMI with TC risk in women (OR = 1.2), excluding males [22]. In a similar vein, a case-control study revealed that excess weight (BMI > 25 kg/m2) increased individual susceptibility to DTC. However, this augmented risk was evident in women but not in men [23]. Moreover, a meta-analysis of 141 prospective studies revealed that a 5 kg/m2 increase in BMI was strongly associated with TC (relative risk = 1.14, 95% CI: 1.06–1.23) in both sexes [24]. In another study comprising of a prospective cohort, it was suggested that BMI was not associated with TC incidence.

A comparison of the mean BMI of our patient set pre-surgery, post-surgery and upon follow up indicated that there was no significant difference in patient’s BMI. A borderline significant association was seen when the mean post-surgery and current BMI (on last follow up) were compared. This effort to compare pre- and post- surgery BMI was in light of the frequent observation that thyroidectomized patients commonly report weight gain which persists in spite of weight loss efforts. A retrospective chart review of 120 patients done by Jonklaas et al. did confirm that patients who had undergone thyroidectomy in the previous year, had weight gain in the following year [25]. However, similar to our findings, Weinreb et al. found no significant difference in weight gain as compared to a control group of euthyroid patients [26].

There are several possible mechanisms reported to explain the relation of TC with obesity. These include insulin resistance (IR), insulin growth factor-1 (IGF-1), adipocytokines, thyroid stimulating hormone (TSH), and estrogens [15]. IR possibly plays a major role as it is associated with other risk factors including IGF-1, adipocytokines, and TSH in a true relationship with a potential for development and/or progression of TC [27]. Increased BMI has also been associated with higher serum TSH values which are shown to be an independent factor for TC. DTCs express TSH receptors on their cell membranes. Higher levels of serum TSH are found to be associated with increased cell proliferation and probably increased risk for mutations and development of TC [28], which are likely to be mediated by TSH receptors [29].

TC occurs more commonly in women as compared to men [23]. This was supported by our study in which the vast majority of patients were female at 61.5%. This gender prevalence can be due to endogenous sex hormone i.e., estrogen. Adipose tissue plays a role in the synthesis and conversion of sex steroids and this role becomes more prominent after menopause when the function of ovaries ceases. Therefore postmenopausal obese women have higher levels of serum estrogen than normal weight women. Estrogen receptors (ER) are expressed on DTC cell lineages and may have modulatory effects on TC cells [30]. However, the preoperative and postoperative mean BMI as investigated in this work, were not significantly different between the two genders.

Several limitations of our study are considered. These include the use of only cases and not controls, due to which the incidence of TC in Pakistan cannot be judged. Also, due to the lack of critical information or consent, we could not include data of all TC patients treated at our hospital. The duration of data collection was also long and certain lifestyle and dietary changes in the subjects have occurred in this time period which our study does not specifically discuss. As documented by Pellegriti et al. in a review of TC risk factors, diet, and lifestyle changes have not been studied in much detail in terms of TC and existing studies have provided controversial results [1].

However, no such information existed in the context of Pakistan before and we report that at a single tertiary care centre these associations of BMI and TC exist and our findings can be generalized to other tertiary care centres of Pakistan.

## Conclusions

This study revealed the associations of TC and BMI in patients presenting to a tertiary care centre in Pakistan. Moreover, TC patients mostly presented with BMI >25 and were categorized as obese according to the WHO standards for Asian populations. Age was significantly associated with the risk of having a higher BMI and all patients (irrespective of gender) above the age of 45 years were obese in this study. Moreover, differences in BMI pre- and post-surgery could not be statistically proven. Further molecular investigations and case-control studies might explain the relationship of BMI and TC in more detail.
